# The Leger-Dorez System of Adjustable Bridge Attachments

**Published:** 1914-05-15

**Authors:** Joseph Nolin

**Affiliations:** Montreal, Canada


### THE LEGER-DOREZ SYSTEM OF ADJUSTABLE BRIDGE ATTACHMENTS.

BY JOSEPH NOLIN, D.D.S., MONTREAL, CANADA.

Bridgework has proved itself such a useful, such a precious auxiliary for the restoration of impaired masticatory organs, that the tendency of late years has rather been to cut away more and more sound material, as it was gradually found that, by so doing, better results could be obtained.

I am an enthusiast and a teacher of crown and bridgework, and, as such, I fully appreciate the services that bridgework, good bridgework, has rendered to the dental profession and to humanity. Still, can it be said truthfully, that bridgework, in its present shape, is all that could be desired, and that it leaves no room for improvements?

Like all human things it has great qualities and great drawbacks. We overlook the latter in view of the former, thus living up to the old saying, that half a loaf is better than none. It is, however, wise to keep these drawbacks ever present in our minds, if for no other reason than to correct them, gradually, as we advance step by step, towards a better knowledge of the fundamental principles of our profession.

The cutting away of sound material, to the greater discomfort, as stated above, of both patient and operator, is one drawback. The difficulty of removing abutment crowns, when it is found necessary to do so, is another.

These two drawbacks, a clever Frenchman, Dr. Leger-Dorez, of Paris, has tried to overcome, with a measure of success which calls for a better recognition of his work on this side of the Atlantic. He has also endeavored, and successfully so, to construct bridges which would leave to the abutments their natural mobility in the socket, making for a better preservation of the physiological conditions of both root, socket and peridental ligaments; thus overcoming a third and perhaps more important drawback.

### The Split Ferule.

"It may appear parodoxical," says Dr. Leger-Dorez, "to say that one may girdle intimately a tooth with gold, without having previously ground off the bulging surfaces, and still be able to obtain perfect adherence of the band and perfect adaptation at the neck of the tooth.

"This is however what has been realized after years of patient study. An example may save a lot of explaining:

"If we would bind a tree with a hoop of iron, without cutting off the branches, we must, if the tree is large, use two half-collars of iron or steel bolted together. If the tree is small we may use a split hoop sprung around the trunk, and securely bolted on one side." It is this principle which has been applied to bridgework and which has proved successful.

The simplest form of the Leger-Dorez bridge (which shall be the only one discussed in this paper) was described by him before the Dental Society of Paris in 1908. The invention was received with varied appreciation. The possibilities of cast work were not then so fully recognized as they are to-day. The theories enunciated were so new, that the inventor's announcement of successful operations in this line of work were received with scepticism by many. As paper followed paper accompanied by models and corroborated clinical applications, the profession had soon to take notice; so much so that to-day dentists from all over Europe flock to Dr. Leger-Dorez's office for further knowledge. The demand for information has been so great in fact that a post-graduate course had to be started in Paris, for the teaching of this particular line of work.

The Leger-Dorez bridge in its simplest form consists of two split bands with tenant extentions, sprung around the abutment teeth; a cast bridge, morticed at both ends; and two locking pins.

### Technique.

The first operation, in the construction of a split ferule bridge, is the taking of the bite.

This is done by getting the patient to bite correctly in a piece of softened wax which is then chilled and carefully removed.

Secondly, a perfect plaster impression is taken, a model is cast and articulated.

Thirdly, the abutment teeth of the bridge are carefully oiled and covered on all their surface, except the occluding one, with a coating of inlay wax. The wax is brought over the angles of the occluding surface as far only as the occlusion of the antagonizing teeth will allow.

This coating of wax is then carefully shaped and smoothed to an even thickness of about 30 gauge. On the side of the missing teeth, an extention assuming the shape of a cuboid tenant is added.

A platinum-iridium pin is now prepared and slightly tapered. This being oiled and heated, is passed through the centre of the wax tenant bucco-labialy.

A vertical incision is now made, with a very thin saw, mesio-distally or vice versa, through the centre of the wax extention and the wax ferule down to the plaster tooth, taking care however not to injure the latter.

Before this operation however the platinum-iridium pin has been removed, leaving a clear hole in the wax extention and is again replaced after the incision. This is to remove any shavings which may obstruct the hole.

If all the above details have been carefully attended to the wax ferule can now be sprung open, removed from the model and invested, the sprue wire being placed at some neutral point. The piece may be cast with coin or platinized gold.

We thus obtain a cast gold split ferule, that should fit almost correctly (more so, in fact, than any soldered ferule could fit) over the abutment tooth.

The surface next to the tooth may then be cleaned and imperfections due to casting accidents, such as air-bubbles and so on, should be carefully corrected. If the contraction of the gold should prevent the two ends of the ferule from coming in perfect contact, when the latter is placed over the tooth, a small piece of 22k gold carefully adjusted and soldered to one side of the gap will easily overcome this accident.

The above technique can evidently not be followed when the abutment teeth is in contact with an adjoining tooth as it would then be impossible to either make, or remove the wax ferule, or to insert a gold ferule in the mouth without first altering the shape of the abutment tooth or its neighbor.

This difficulty is overcome by filing down the contact point of the abutment tooth to the thickness of a 30 gauge piece of gold plate.

A triangular piece of 22k gold, through which three or more holes have been punched, is fitted into place. The holes are filled with wax and the surface next to the tooth is covered with a thin coating of the same material.

This is placed into position in the mouth before taking the impression and can be easily loosened from the model by heating it. Any trace of wax is removed from the plaster model, which is oiled as before. The piece of gold recoated with wax is placed back into place, forming the approximal side of the ferule, which is completed in wax as heretofore described. The piece of gold should, buccally and lingually, overlap the wax ferule so as to become thoroughly imbedded in the investment.

### The Bridge.

The cast bands, having been "tried" in the mouth and corrected, are now placed on the model, closed with narrow pliers and locked by inserting the platinum-iridium pin through the hole left in the tenant extention. This hole has been corrected or redrilled if found necessary. The pin should fit correctly in the hole, but not so tightly however as to be difficult of removal.

If the insertion of the pins should not hold the ends of the ferule in perfect contact, a small hole may be perforated through the two parts of the split extention, and these tightly tied together with a silk ligature.

The gold band, the extention and the platinum pins are oiled. Melted inlay wax is poured over the pegs and the extention. A bridge of wax is built across, from one abutment to the other, carved and articulated as any ordinary cast bridge, and chilled. The pins are then withdrawn and the wax body carefully removed, invested and cast.

When porcelain facings are to be used their lingual surface should be oiled, and they should be backed with wax, to a thickness corresponding to the length of the platinum pins (which insures a better adaptation of the wax to the porcelain), the facings removed before investment, and graphite points inserted in the holes left by the pins. The holes left at each end of the wax bridge by the pins may also be filled with graphite, although it will be found that if the holes are perforated through and through and carefully filled with investing material, good results can be obtained, provided the investment is carefully handled while heating, and during the casting operation. The bridge after being cleaned, finished and polished is ready for insertion in the mouth.

### Adjustment and Correction.

The split ferules are placed in the mouth, pressed home with convenient pliers and the bridge is put into place by slipping the mortice like openings of the bridge over the tenant shaped extention of the abutments. The locking pins are slipped into place and the completed apparatus is examined, the occlusion being carefully revised, and any necessary corrections made. The whole is then ready for the final operation of cementation.

The teeth are cleaned and dried. Soft cement is spread over the inside surface of the ferules and the latter are sprung into their normal position over the abutment teeth. With narrow flat-nosed pliers the two halves of each tenant are firmly but delicately pressed together. Strong silk ligatures which have been previously passed through holes, bored for this purpose through the extentions, are quickly tied and the body of the bridge slipped into place and pressed home. After sufficient time has been allowed for the complete setting of the cement the body of the bridge is removed. The mortice-like depressions at each end of the gingival surface of the bridge and the tenant-shaped extensions of the ferules are covered with a thin coating of cement and the bridge is quickly inserted into place. Before the cement has had time to harden the platinum-iridium pegs are quickly smeared with cement and inserted. The protruding ends of the pegs may now be ground and polished and the operation is completed.

Dr. Leger-Dorez has improved his technique in several ways, since giving out to the dental profession his interesting invention.

He has for instance applied his eye and pin-locking system to the crowning of crownless teeth. His split ferule, he now securely locks into place by means of a screw arrangement, which binds together the two halves of the tenant attachment, making for greater ease in cementation and giving greater security to the ferule.

A resume of the principal advantages of the Leger-Dorez System of Split Ferule Crowns, and Interlocking Bridges would be as follows:

1. The saving of sound tooth material.

2. The saving of pain to the patient and of fatigue and discomfort to both patient and operator.

3. Ease in removing and replacing bridges without injury to either the bridge itself or the abutments.

4. Greater facility in bridging converging teeth or teeth with extra long interlocking cusps.

5. Better restoration of the masticatory functions through allowing the teeth to retain their physiological motion in the sockets.

You will be all the more willing to join me, I am sure, in thanking and congratulating Dr. Leger-Dorez for his invention when I say that, differing in this from some other dental inventors he has, from the very start, taken the profession into his confidence; revealing, step by step, through society papers and the French dental reviews, the progress of his discoveries. In failing, moreover, to patent any of his appliances or to place on the market a set of very highly nickeled, very costly and (at times) very useless instruments, he has proven himself to be what is more than a great inventor or a great mechanical genius—a real professional man.—*Oral Health.*

